# Assessing body awareness and upper extremity functionality in breast cancer survivors with and without lymphedema: a comparative analysis with healthy controls

**DOI:** 10.1007/s00520-024-09138-2

**Published:** 2025-01-10

**Authors:** Cansu Şahbaz Pirinççi, Emine Cihan, Fatıma Yaman

**Affiliations:** 1 Gulhane Faculty of Physiotherapy and Rehabilitation, University of Health Sciences, Ankara, Turkey; 2 Department of Therapy and Rehabilitation, Vocational School of Health Sciences, Selcuk University, Konya, Turkey; 3 Department of Physical Medicine and Rehabilitation, Faculty of Medicine, Kutahya Health Sciences University, Kutahya, Turkey

**Keywords:** Breast cancer, Lymphedema, Body awareness, Upper extremity

## Abstract

**Purpose:**

To determine whether body awareness and upper extremity functionality are affected in patients with or without lymphedema development after breast cancer surgery (BCS) in comparison with individuals without a history of cancer.

**Methods:**

The study included a total of 102 individuals, including 34 who developed lymphedema after BCS (mean age: 43.88 ± 12.13 years), 34 who did not develop lymphedema after BCS (age: 44.67 ± 11.20 years), and 34 without a history of any cancer surgery (age: 45.41 ± 12.13 years). The participants’ demographic data were recorded. Body awareness was evaluated using the Body Awareness Questionnaire, and upper extremity functionality was evaluated using the Quick-Disabilities of the Arm, Shoulder, and Hand questionnaire.

**Results:**

The demographic data of the groups were similar (*p* > *0.05*). While there were differences between the BCS groups in terms of operative time (*p* < *0.001*) and operated breast (*p* = *0.001*), the number of lymph nodes dissected and the type of surgery performed were homogeneously distributed (*p* > *0.05*). Body awareness and upper extremity functionality were significantly lower in the lymphedema group than in the BCS group without lymphedema and the control group (*p* = *0.021* and *p* < *0.001*, respectively).

**Conclusion:**

The development of upper extremity lymphedema after BCS adversely affects both body awareness and upper extremity functionality.

**Supplementary Information:**

The online version contains supplementary material available at 10.1007/s00520-024-09138-2.

## Introduction

Breast cancer is one of the types of neoplasms with the highest fatality rate in women. Despite the benefits of diagnosis and treatment, this process negatively affects women’s well-being. Recent statistics indicate that breast cancer has surpassed lung cancer, which was previously the most common cancer in the world, with approximately 2.3 million new cases [1]. As side effects of treatment, changes in physical appearance, especially hair loss and deformities, cause women to feel “inadequate” [2]. Lymphedema caused by damage to the lymphatic system during cancer treatment can further complicate the condition of many patients. Emotional effects gradually increase with skin changes, fibrosis, loss of sensation, a sense of heaviness in the extremity, a feeling of fullness, deformity, and functional deficiencies [3].

Lymphedema is the accumulation of protein-rich tissue in the interstitial space. Dysfunction of lymphatic structures disrupts the drainage of the lymphatic system. The main destructive aspect of this process is the appearance of the limb, which causes morbidity. Although many factors are involved in the etiology of lymphedema, it most commonly occurs after breast cancer [4]. Lymphedema causes heaviness, numbness, and pain in the upper extremity and limits the range of motion of the joint, causing dysfunction in the upper extremity. When psychological effects are added to loss of function, patients may experience negative emotions, such as loss of confidence, frustration, anxiety, and increased self-consciousness [5].

After BCS, there may be negative effects on patients' body awareness due to reasons such as the type of surgery, surgical scars, skin burns after radiotherapy, hair loss as a side effect of chemotherapy, and weight change [6, 7]. In addition, the changing extremity shape due to lymphedema, which is a common change in the extremity after BCS, may cause parameters such as self-perception and body awareness to be negatively affected [8]. However, within the scope of our research, studies that hypothesize body awareness and upper extremity functions in patients who develop lymphedema in the upper extremity after BCS and BCS are limited.

In light of this need, in this study, we aimed to investigate body awareness and upper extremity functions in patients diagnosed with lymphedema after breast cancer surgery (BCS) and to cast light on their rehabilitation process. Our hypotesis is “in individuals who develop lymphedema after BCS, a significant decrease in body awareness and upper extremity functionality is expected when compared to individuals without lymphedema and healthy controls.”

## Methods

### Study design and participants

The study was conducted at Kutahya Health Sciences University Evliya Celebi Training and Research Hospital between August and October 2023. Before commencing the study, ethical approval was obtained from the local ethics committee of Kutahya Health Sciences University Faculty of Medicine (ethic number:2023/09–34, date:16.08.2023), and the study was conducted in accordance with the tenets of the Declaration of Helsinki.

G*Power software package (G*Power, Version 3.0.10, Franz Faul, Universität Kiel, Germany) was used to calculate the sample size. The primary finding of the study was the body awareness.Fifteen participants from each group were randomly recruited for the pilot study. The effect size corresponding to this value was 0.40. It was calculated that in order to achieve 90% power with α = 0.05 type I error, 28 patients were required for each group. When this value was increased by 20 percent in order not to decrease the study power due to possible dropout, it was seen that at least 34 patients were required for each group.

In the study, a total of 115 participants were consulted, including 42 who had undergone BCS, 36 who developed upper extremity lymphedema after the BCS, and 37 controls. Four patients who had BCS declined to participate, 3 patients had undergone shoulder surgery, and 1 patient was excluded due to metastasis. Two patients with stage 1 lymphedema after BCS were not included in the study. In the control group, 3 individuals were excluded because they refused to participate. The sample included 102 individuals, including 34 who developed lymphedema after BCS, 34 who did not develop lymphedema after BCS, and 34 who did not have a history of any cancer surgery.

The inclusion criteria for the lymphedema group were as follows: being woman, being aged 18–65 years, having undergone unilateral BCS, being diagnosed with stage 2 or 3 secondary lymphedema in the upper extremity according to the International Lymphedema Association [9], if a mastectomy has been performed, using a breast prosthesis after the surgery, not having any orthopedic disease that would prevent walking, who had not undergone upper extremity surgery, volunteering to participate in the study. The inclusion criteria for the BCS group without lymphedema were being female, being aged 18–65 years, having undergone unilateral BCS, who had not undergone upper extremity surgery, volunteering to participate in the study.

The control group consisted of individuals from the same age range as the participants in the study group, including volunteers among patient escorts, hospital staff, or individuals who came for general health check-ups. The inclusion criteria for the control group were being female, being aged 18–65 years, having no history of cancer surgery, who had not undergone upper extremity surgery, volunteering to participate in the study. In addition, the following exclusion criteria were used in all groups: not being willing to participate in the study; the presence of metastases, neurological, or orthopedic disorders; postural deformities, such as scoliosis and kyphosis; mental and cognitive disorders; or communication/cooperation problems, having received physical therapy for the upper extremity in the last 6 months, having a history of local injections for the upper extremity in the last 3 months, having shoulder pathology such as frozen shoulder, impingement etc., having systemic diseases rheumatoid arthritis, diabetes etc., having chronic diseases such as hypertension, copd, fibromyalgia, chronic depression, those who have had cancer rehabilitation before.

### Data collection and outcome measures

All participants were informed before the study. Evaluations were undertaken face-to-face. The participants’ sociodemographic and clinical characteristics¸ including age, height, weight, body mass index (BMI) and dominant extremity were recorded. In addition, for the patients in the BCS groups, the type of operation, time since operation (months), operated breast, and number of dissected lymph nodes were noted. In all participants, body awareness was evaluated using the Body Awareness Questionnaire, and upper extremity functionality was evaluated using the Quick-Disabilities of the Arm, Shoulder, and Hand questionnaire.

Body awareness was assessed using the Turkish version of the Body Awareness Questionnaire, which has been previously reported to be valid. This instrument consists of a total of 18 items evaluated based on a scale of 1 (not at all true of me) to 7 (very true of me). The total score is obtained by adding the scores of each item. A higher total score indicates better body awareness. The test–retest reliability coefficient was 0.830 at 3-day intervals. Cronbach's alpha was calculated as 0.917 to determine internal consistency [10].

Upper extremity functionality was assessed using the Quick-Disabilities of the Arm, Shoulder, and Hand questionnaire. The validity of the Turkish version of this questionnaire has been previously reported. Each of the 11 items in this instrument is scored from 1 to 5. The responses given to all items are summed up, and the total score is obtained with a special formula. A high total score indicates a high level of functional limitation. Test–retest reliability of DASH-Function/Symptom was 0.910. It was concluded that the Turkish version of the DASH Questionnaire has excellent test–retest reliability and validity and is an adequate and useful tool for measuring functional disability in upper extremity complaints in Turkish-speaking patients [11].

### Statistical analysis

The data were analyzed using the SPSS v. 19.0 software package. Continuous variables were expressed as mean ± standard deviation, and categorical variables as numbers and percentages. The Shapiro–Wilk test was used to examine the conformity of the variables to the normal distribution. The one-way analysis of variance test was used for parametric variables, and the Kruskal–Wallis test was used for non-parametric variables to analyze differences between groups. The Mann–Whitney U test was used to utilized to test the differences between the groups. A post-hoc analysis and the Bonferroni correction were undertaken to determine the group(s) causing the significant difference. The analysis of categorical variables was performed with the chi-square test. The significance level was accepted as *p* < *0.05*.

## Results

The mean age of the participants was 45.41 ± 12.13 years for the control group, 44.67 ± 11.20 years for the BCS group without lymphedema, and 43.88 ± 12.13 years for the BCS group with lymphedema. There was no significant difference between the three groups in terms of age (*p* = *0.883*), BMI (*p* = *0.349*) and dominant extremity (*p* = *0.490*) (Table 1).

**Table 1:** Demographic data of the participants

	Control group		BCS group without lymphedema		BCS group with lymphedema			
	(*n* = 34)		(*n* = 34)		(*n* = 34)			
Parameter	X ± SD	Median (min–max)	X ± SD	Median (min–max)	X ± SD	Median (min–max)	x^2^	*p*
Age (years)	45.41 ± 12.13	50 (25–65)	44.67 ± 11.20	43 (25–65)	43.88 ± 12.13	42 (20–65)	0.249	*0.883*
Height (cm)	162.82 ± 7.42	162.5 (150–182)	160.44 ± 5.42	160 (145–170)	161.14 ± 5.48	160 (150–176)	1.431	*0.489*
Weight (kg)	69.11 ± 15.66	67.5 (50–115)	70.94 ± 9.16	73.5 (52–85)	69.52 ± 12.58	70.5 (51–100)	1.497	*0.473*
BMI (kg/m^2^)	26.06 ± 5.34	26.41 (17.71–37.77)	27.65 ± 4.10	28.25 (19.36–39.95)	26.91 ± 5.39	27.28 (17.85–40.05)	2.107	*0.349*

*BCS* breast cancer surgery, *X* mean, *SD* standard deviation, *n* number of participants, *min* minimum, *max* maximum, *x*^*2*^ Kruskal–Wallis test or chi-square test statistic, *p* < *0.05*

There was a significant difference between the patients with and without lymphedema development after BCS groups in terms of operative time (*p* < *0.001*) and operated breast (*p* = *0.001*), but the number of dissected lymph nodes (*p* = *0.605*) and the type of surgery (p = 0.051) were similar (*p* = *0.605*). Of the patients who developed lymphedema, 58.8% had stage 2 and 41.2% had stage 3 lymphedema (Table 2).

**Table 2:** Medical histories of patients with and without lymphedema development after BCS

		BCS group without lymphedema (*n* = 34)		BCS group with lymphedema (*n* = 34)			
Parameter		X ± SD	Median (min–max)	X ± SD	Median (min–max)	z	*p*
Operative time (months)		3.11 ± 2.23	3 (1–12)	30.52 ± 28.95	24 (6–120)	−7.051	** < *0.001***
Number of dissected lymph nodes		8.50 ± 6.32	7 (1–25)	9.32 ± 8.00	7.5 (1–40)	−0.517	*0.605*
		***n***	**%**	***n***	**%**	**x**^**2**^	***p***
Operated breast	Right	10	29.4	24	70.6	11.529	***0.001***
	Sol	24	70.6	10	29.4		
Type of surgery	Lumpectomy	19	55.9	11	32.4	3.818	*0.051*
	Modified radical mastectomy	15	44.1	23	67.6		
Lymphedema stage	2	-	-	20	58.8	-	-
	3	-	-	14	41.2		

*BCS* breast cancer surgery, *X* mean, *SD* standard deviation, *n* number of participants, *min* minimum, *max* maximum, *z* Mann–Whitney U test statistic, *x*^*2*^ chi-square test statistic, *p* < *0.05*

Among the assessment parameters, body awareness significantly differed between the three groups (*p* = *0.024*). This significance resulted from the comparison of the control group and lymphedema groups (*p* = *0.021*). Significant differences were also found when upper extremity functionality was compared between the three groups (*p* < *0.001*). Upper extremity functionality significantly differed between the control group and the lymphedema group (*p* < *0.001*), as well as between the patients with and without lymphedema development after BCS (*p* = *0.026*) (Table 3).

**Table 3:** Comparison of assessment parameters between the groups

	Control group (1)		BCS group without lymphedema (2)		BCS group with lymphedema (3)					
Parameter	X ± SD	Median (min–max)	X ± SD	Median (min–max)	X ± SD	Median (min–max)	F	*p*	Comparison	*p*
Body awareness	106.85 ± 6.96	94–118	100.94 ± 16.80	60–125	97.38 ± 16.58	61–120	3.852	***0.024***	1 vs. 2	*0.268*
									1 vs. 3	***0.021***
									2 vs. 3	*0.913*
Upper extremity functionality	16.64 ± 11.74	0–47.73	26.14 ± 18.63	2–72.5	36.90 ± 18.47	2.27–77.2	12.681	** < *0.001***	1 vs. 2	*0.061*
									1 vs. 3	** < *0.001***
									2 vs. 3	***0.026***

*BCS* breast cancer surgery, *X* mean, *SD* standard deviation, *min* minimum, *max* maximum, *F* one-way analysis of variance test, *p* < *0.05*

According to lymphedema stages, body awareness and upper extremity functionality are similar between groups (Table 4).

**Table 4:** Comparison of body awareness and upper extremity functionality in patients with lymphedema according to disease stage

	Stage 2		Stage 3			
	X ± SD	Median (min–max)	X ± SD	Median (min–max)	z	*p*
Body awareness	97.35 ± 18.49	99 (61–120)	97.42 ± 14.07	94.50 (76–120)	0.333	*0.739*
Upper Extremity Functionality	35.51 ± 18.09	32.50 (2.27–77.20)	38.88 ± 19.51	36.70 (18.18–77.20)	0.247	*0.805*

*X* mean, *SD* standard deviation, *min* minimum, *max* maximum, *z* Mann–Whitney U test, *p* < *0.05*

## Discussion

The demographic data of the participants in the study were similar, but the accompanying diseases and the operated breast differed. Body awareness was lower in the lymphedema group than in the remaining two groups. There were also differences between the groups in terms of upper extremity functionality. Body awareness and upper extremity functionality did not significantly differ according to disease stage in the lymphedema group.

Body awareness is one of the parameters affected by physical and psychological changes after BCS. Factors such as the type of surgery performed, scars caused by surgery, changes in shoulder mobility, skin color changes caused by radiation therapy, and the side effects of chemotherapy can alter the body image of patients [12, 13]. In a previous study, it was found that body image changed positively with a decrease in the risk of mortality [14]. The development of lymphedema after BCS causes functional impairment and is a reminder of breast cancer treatment. Lymphedema, a chronic condition that is difficult to conceal, can affect individuals’ body image [13]. In a study investigating the body image of patients who developed lymphedema and those who had a history of any trauma, it was determined that the lymphedema group had lower body awareness in seven of the nine sub-parameters of the questionnaire used for evaluation, compared to the trauma group [15]. In another study, body image was found to be an important factor in understanding depressive symptoms in patients who developed lymphedema in the upper extremity after BCS, and the authors recommended examining the pain levels and body images of patients suffering from lymphedema [3]. Similarly, Hoyle et al. found body image and sexual concerns to be intense and disturbing among patients who developed lymphedema after BCS [16]. In the current study, it was determined that patients who developed lymphedema in the upper extremity BCS had lower body awareness than those who did not develop lymphedema after this surgery and healthy controls. However, the presence of a history of BCS did not affect body awareness.

Upper extremity functionality is one of the most affected parameters after BCS. Both BCS and chemotherapy/radiotherapy applications cause muscle strength to be negatively affected, especially in extremity movements, and this series of problems can lead to the restriction of activities, such as carrying objects and wearing clothes [17]. In a previous study, it was reported that radiotherapy after BCS adversely affected the pectoral muscles and resulted in a 10% restriction in normal joint movement compared to the unaffected extremity [18]. In our study, the upper extremity functionality of the BCS group without lymphedema was found to be higher than that of the control group. In the literature, it has been reported that the development of lymphedema in the operated extremity causes adverse effects on the scapular muscles as well as the pectoral muscles [19]. In a study comparing the upper extremity functionality of patients with and without lymphedema development after BCS, Smoot et al. observed that those who developed lymphedema had worse upper extremity functionality [20]. Similarly, in our study, the upper extremity functionality of the group with lymphedema was found to be lower than both the BCS group without lymphedema and the control group.

There are some limitations to our study. First, we included both patients who underwent lumpectomy and those who underwent modified radical mastectomy in the sample. Since the breast is affected differently by these surgical techniques, the degree of their negative impacts on body image may differ. However, we did not question whether patients who had undergone a modified radical mastectomy used breast prostheses. The presence of an artificial limb can also change functionality and affect body image. Second, only the functionality of the participants was evaluated, although body image also includes psychological parameters that should also be assessed. It is recommended that future studies take these limitations into account. The difference in evaluation timing between the two groups could influence the results related to body awareness and upper limb functions. This timing discrepancy might introduce variability, potentially affecting the comparability of the data. To reduce this limitation, future studies should aim to conduct evaluations at the same postoperative time point for all participants, ensuring more consistent and reliable results.

## Conclusion

Although BCS does not adversely affect body awareness, the development of lymphedema in the extremities has negative effects on the body awareness of patients. However, we determined that the stage of lymphedema was not an effective parameter for body awareness and that the development of lymphedema alone was sufficient to affect body image. Furthermore, we observed that upper extremity functionality was not adversely affected by BCS, but this negative effect emerged with the development of lymphedema. Body awareness should be closely monitored in patients who have undergone BCS and develop lymphedema, to ensure timely and effective management of potential functional impairments. Early intervention and tailored rehabilitation strategies should be considered to mitigate these adverse effects.

## Supplementary Information

Below is the link to the electronic supplementary material.

Supplementary file1 (DOCX 608 KB)

## Data Availability

Not applicable.
