# Supplementary material for: Assessing body awareness and upper extremity functionality in breast cancer survivors with and without lymphedema: a comparative analysis with healthy controls
Source: Support Care Cancer. 2025 Jan 10;33(2):86. doi: 10.1007/s00520-024-09138-2 (PMC11717817; doi:10.1007/s00520-024-09138-2)

Turkish version of body awareness questionnaire


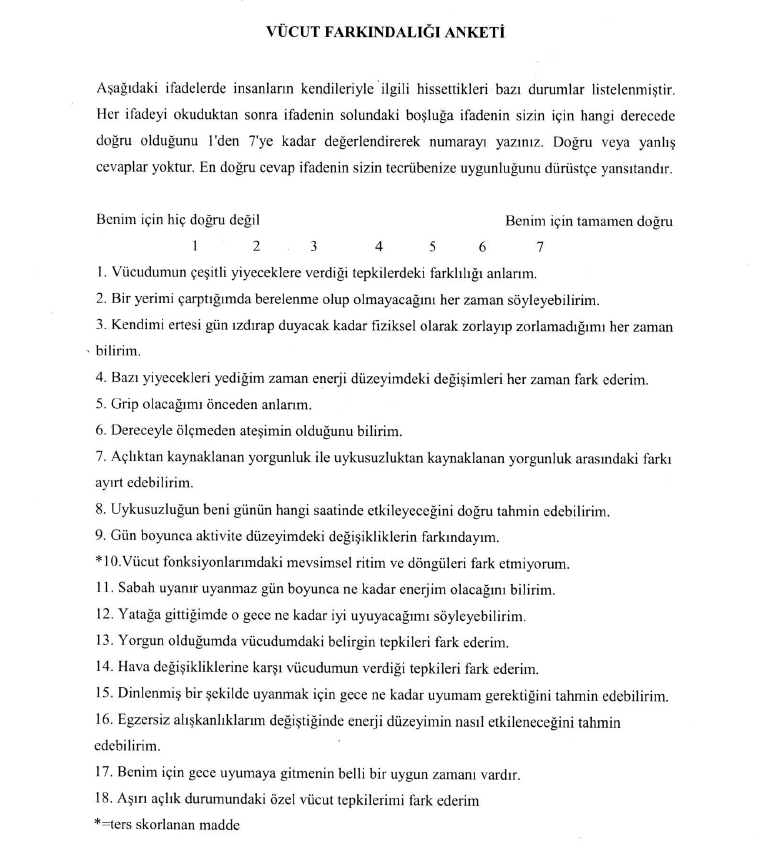


Turkish version of Quick-Disabilities of the Arm, Shoulder, and Hand questionnaire


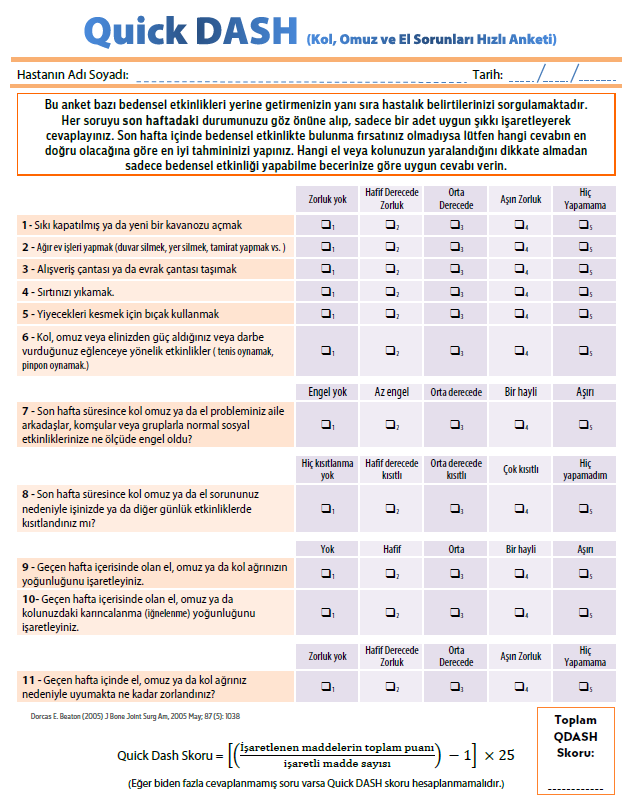

Supplement: Supplementary file 1 — Supplementary file1 (DOCX 608 KB) [file 520_2024_9138_MOESM1_ESM.docx]
